# Importance of S‑Doped Porous Carbon Acidity and Visible Light Photoactivity for Its Antibacterial Activity

**DOI:** 10.1021/acsami.5c14239

**Published:** 2025-11-04

**Authors:** Danela Sadikaj, Abanob Fekri, Isabelle Bautista, Nafisatu Davis, Tasnim Agha Alkla, Muhammad Moueed Haider Mirza, Jiaying Wang, Phillip Stallworth, Natalie Hudson-Smith, Teresa J. Bandosz, Xiaojun Yu, Steven Greenbaum, Wanlu Li

**Affiliations:** † Department of Earth and Environmental Science, 8087 Montclair State University, Montclair, New Jersey 07043, United States; ‡ Department of Chemistry, Saint Peter’s University, Jersey, New Jersey 07306, United States; § Department of Chemistry and Biochemistry, 8087 Montclair State University, Montclair, New Jersey 07043, United States; ∥ Department of Biomedical Engineering, 33694 Steven Institute of Technology, Hoboken, New Jersey 07030, United States; ⊥ Department of Physics and Astronomy, 5924 Hunter College, New York, New York 10065, United States; # Department of Chemistry and Biochemistry, 14770 City College of New York, New York, New York 10031, United States; ∇ New Jersey Center for Water Science and Technology, Montclair State University, Montclair, New Jersey 07043, United States; ▲ Department of Theoretical Chemistry, Institute of Chemical Sciences, Faculty of Chemistry, Maria Curie-Skłodowska University, Lublin 20-031, Poland

**Keywords:** sulfur-doped porous carbon, photocurrent, disinfection, *E. coli*, holes

## Abstract

Bactericidal activity of sulfur-doped carbon has been investigated against a common Gram-negative bacterium, *Escherichia coli* K-12 (*E. coli*), in aqueous solution. Under dark conditions, 1 h contact of carbon with bacteria resulted in a 3–4 log decrease from the initial concentration of 7 log. When exposed to visible light, a 6–7 log decrease in *E. coli* was observed. The bactericidal activity under dark conditions was linked to the effects of functional groups, particularly sulfonic groups, which released protons that contributed to bacterial death. Even though under visible light no reactive oxygen species (ROS) were detected in electron paramagnetic resonance (EPR) and scavenger experiments, the enhanced bactericidal activity was due to the generation of holes in the carbon matrix. The photoactivity of sulfur-doped carbon is attributed to hole generation in aqueous solution under visible light, further enhancing its bactericidal effect. Bacterial death was not affected by the surface area or the porous structure of carbons.

## Graphical abstract

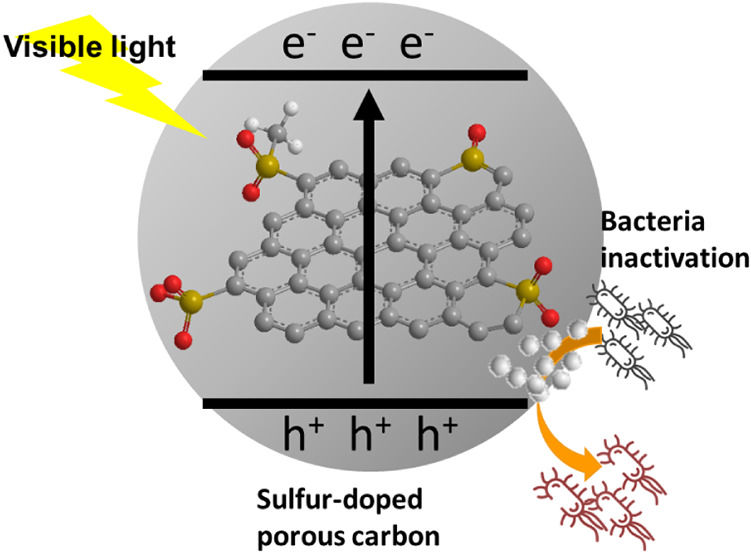

## 1 Introduction

Various carbon allotropes, including graphene-based nanomaterials,^1–6^ carbon nanotubes,^7,8^ and carbon quantum dots,^9–15^ have been studied as antibacterial materials. As for graphene-based nanomaterials, two essential characteristics were found to influence their antibacterial activity: size and the density of oxygen-containing functional groups (referred to by some authors as “oxidation capacity”).^1^ Chen’s group systematically evaluated the antibacterial activity of graphite (Gt), graphite oxide (GtO), graphene oxide (GO), and reduced graphene oxide (rGO). GO exhibited the highest antibacterial activity, followed in order by rGO, Gt, and GtO. In the viability test, GO showed the maximum activity against *E. coli*. GO dispersion at a concentration of 80 μg/mL killed 91.6% *E. coli* (about a 1 log reduction) after 4 h of contact. Scanning electron microscopy images indicated that most of the cell membranes were disrupted by the GO and rGO nanosheets. Samples with higher “oxidation capacity” were able to induce oxidative stress in bacterial cells, and smaller sizes led to higher antibacterial activity. Hu et al. demonstrated that GO at a concentration of 85 μg/mL significantly slowed down the growth of *E. coli*, leading to a viability loss of up to 98.5% (nearly a 2 log reduction).^2^ Transmission electron microscopy (TEM) images revealed that the cell membrane of *E. coli* was severely damaged by either oxidative stress or physical disruption after being exposed to GO. Another study showed that the position of bacterial attachment depends on the type of graphene material.^3^ Bacteria were found to be distributed over the GO surface, while for pristine graphene and rGO flakes, bacteria were attached to their edges. In that study, the interaction between bacteria and materials was investigated without shaking, eliminating a size/shape effect.

To the best of our knowledge, only one study focused on the GO antibacterial activity under simulated sunlight.^16^ Among all of the reactive oxygen species (ROS), singlet oxygen (^1^O_2_) was detected by GO through electron paramagnetic resonance (EPR). Nevertheless, the GO-mediated oxidative stress was found to be ROS-independent. EPR analysis also confirmed the presence of light-induced electron–hole pairs. The antioxidant glutathione was oxidized upon light in the presence of GO from the electron–hole pairs generated on the surface of GO. The studies used ascorbic acid and glutathione as probes to confirm that carbon-centered free radicals oxidized them. Additionally, light-induced electrons were found to promote the reduction of GO.

In contrast to graphene-based materials that often display strong antibacterial effects, porous carbons have been shown to provide favorable sites for microbial adhesion, colonization, and growth.^17–20^ The interactions between bacteria and porous carbons have started to receive attention since the 1980s. Porous carbon acts as a neutralizer of stress compounds in solution. The porous surface of the carbon can provide a protective environment against fluid shear forces.^21^ As a result, the bacteria attached to the carbon surface are highly resistant to disinfection.^17,22^ Johansson’s group monitored a microbial population between influent and effluent waters of the granular activated carbon columns in a pilot water treatment plant. A higher population of microbes was detected in the effluent waters, indicating microbial growth in the carbon column. In addition, the surface chemistry and porous structure also affect how bacteria interact with porous carbon. It is found that the hydrophobic nature and macropore volume of carbon promote bacterial adhesion and adsorption.^23^ Since porous carbons can exhibit a broad range of properties, there is a need to explore how these properties, mainly surface chemistry, could alter their interactions with bacteria.

While conventional porous carbons tend to promote bacterial attachment and growth, surface modification through heteroatom doping may fundamentally alter their interaction with microorganisms. Sulfur-doped porous carbons showed photoactivity under visible light,^24,25^ and this property might affect their disinfection efficiency under these conditions. Previous studies have reported that sulfur-functionalized carbon materials exhibit strong antibacterial activity.^11,26^ Molaei’s group confirmed the generation of ROS from sulfur-functionalized carbon dots using the oxygen radical absorbance capacity method. They reported that the antimicrobial activity of sulfur-doped carbon (S-CD) is attributed to surface functional groups, such as sulfonate and sulfonic acid, which can interact with the −SH groups of enzymes and induce ROS stress, leading to the denaturation of proteins and lipids.^26^

However, the antibacterial properties of sulfur-doped porous carbons have not been systematically investigated. The objective of this study is to explore the antibacterial activity of sulfur-doped porous carbons with emphasis on the effect of their photoactivity. Fourier transform infrared spectroscopy (FT-IR), thermal analysis, potentiometric titrations, and X-ray photoelectron spectroscopy (XPS) were applied as characterization methods, and they provided detailed information on carbon’s surface features in terms of specific oxygen and sulfur chemical environments. TEM and porosity analysis were performed to show the texture of the carbons. The antibacterial activity was investigated under both dark and visible light conditions. The relationship between the properties of carbons (chemical environments and porosities) and their antibacterial activity is discussed. The mechanisms of *E. coli* killing under light conditions are proposed based on the results from EPR and scavenger experiments. To support the role of specific sulfur species in bactericidal activity, two sulfur-doped carbons with different amounts of sulfur and speciation were tested. By correlating carbon’s surface chemistry and porosity with its antibacterial performance, this work provides new insights into how sulfur speciation influences bactericidal mechanisms.

## 2 Experimental Section

### 2.1 Materials

Poly­(4-styrenesulfonic acid-*co*-maleic acid) sodium salt and poly­(sodium 4-styrenesulfonate) were chosen as polymer precursors for carbons in this study.^27^ C-1 carbon was obtained by carbonization of the poly­(4-styrenesulfonic acid-*co*-maleic acid) sodium salt at 700 °C for 1 h in a horizontal furnace. The nitrogen flow was set at 300 mL/min, and the heating rate was 50 °C/min. C-2 was obtained by carbonization of the poly­(sodium 4-styrenesulfonate) at 550 °C for 1 h with the same settings as C-1. Even though C-2 has been addressed in detail in ref 27. A new batch with intensive washing was obtained and fully characterized in this study. The carbons were washed in a Soxhlet apparatus with water until the leachate reached a stable pH. The carbons were sieved, and a particle size between 44 μm and 106 μm was selected to treat *E. coli*.

### 2.2 Characterizations

#### 2.2.1 FT-IR

FTIR spectra were collected using a Thermo Nicolet Nexus 470 spectrometer using the attenuated total reflectance (ATR) method. They were obtained from a wavelength range of 550–4000 cm^–1^ with 32 scans and a resolution of 4 cm^–1^. The powdered samples were measured without the KBr.

#### 2.2.2 Thermogravimetric Analysis

A thermogravimetric analysis was performed on a Q600 thermal analyzer (TA Instruments). The samples were heated at a rate of 10 °C/min to 1000 °C under N_2_ (100 mL/min).

#### 2.2.3 Potentiometric Titration

Potentiometric titration was conducted by using the 888 Titrando automatic titrator (Metrohm). Approximately 0.05 g of samples was dispersed in 25 mL of 0.100 M NaNO_3_ (Sigma-Aldrich, 99%). Throughout sample preparation and titration, the suspension was stirred with a magnetic stirrer and purged with N_2_ to eliminate the presence of CO_2_. Once the pH of the suspension became stable (reported as the surface pH), a 0.100 M HCl standard solution (Fisher Scientific) was added to the suspension to adjust the pH to ∼3. Subsequently, titration was carried out using a 0.100 M NaOH standard solution (Fisher Scientific) until the pH reached 11. Titration data were used to generate proton binding curves following the method described in ref 28, and p*K*_a_ distributions were calculated based on proton binding curves using the SAIEUS method.^29^

#### 2.2.4 Zeta Potential

To determine the surface zeta potential, 4 mg/mL of carbon was stirred in a saline solution overnight. A zeta potential analyzer (British Malvern Instrument Co., Ltd., Britain) was used to measure the zeta potential of the suspension. The pH of the suspensions was also measured.

#### 2.2.5 XPS

XPS analysis was conducted by using a PHI 5000 VersaProbe II system (Physical Electronics), which utilized a monochromatic Al Kα radiation source (1486.6 eV) operating at 15 kV and 50 W within the analyzer chamber. Spectral deconvolution was carried out using Multipak software with Shirley-type background subtraction applied to all recorded signals. Binding energies were determined by fitting the spectra with a combination of Gaussian–Lorentzian (Gauss-Lorentz) functions.

#### 2.2.6 Porosity

Nitrogen isotherms were obtained on an ASAP 2020 instrument (Micromeritics) at −196 °C. The surface area, *S*_BET_, was calculated using the BET method. Pore size distribution was analyzed using Non-Local Density Functional Theory (NLDFT) as implemented in the SAIEUS software.^29^ NLDFT was also used to determine the volume of micropores (*V*_mic_) and pores with sizes smaller than 1 nm in diameter (*V*_<1nm_). Total pore volume was estimated from the isotherm at a relative pressure *p*/*p*_o_ of 0.98.

#### 2.2.7 TEM Images of Carbon Samples and *E. coli* after Exposure to Samples

TEM images were obtained using a Hitachi H-7500 TEM at a 100 kV operating voltage. Images of samples containing *E. coli* were taken immediately after the antibacterial activity test described in Section 2.3.2.

#### 2.2.8 Photocurrent and Mott–Schottky Plot

Photoelectrochemical experiments were conducted in a three-electrode configuration using 0.5 M Na_2_SO_4_ electrolyte, which had been purged with nitrogen for 20 min. A saturated Ag/AgCl electrode (3 M NaCl) served as the reference, while a platinum wire functioned as the counter electrode. To prepare the working electrode, a slurry composed of carbon and poly­(vinylidene fluoride) (PVDF) in a 90:10 weight ratio, dispersed in *N*-methyl-2-pyrrolidone (NMP), was applied onto a titanium foil substrate with an active area of 1 cm^2^. The electrodes were dried in air at 120 °C. The total mass of the active electrode material was around 5 mg. A solar simulator (LCS-100, Newport) equipped with a 400 nm cutoff filter (20CGA-400) served as the light source for irradiation. Electrochemical measurements were carried out using a VersaSTAT workstation (PMC-200, Princeton Applied Research). Prior to illumination, the system was allowed to reach a stable dark current at the applied potential. Transient photocurrent responses were recorded under open-circuit conditions with periodic light on/off cycles.

#### 2.2.9 EPR Analysis

All EPR measurements were carried out at ambient temperature by using an EPR spectrometer (Magnetech ESR 5000). The spin adducts were detected at a 10 mW microwave power between 1 and 2 G field modulation. For analysis, 50 μL of sample solutions was placed into individual borosilicate capillary tubes (0.9 mm internal diameter), which were then inserted into 4 mm quartz tubes. The spin trap 2,2,6,6-tetramethylpiperidine (TEMP) was used to verify the presence of singlet oxygen generated by carbon upon exposure to visible light from the solar simulator. The 2,2,6,6- tetramethylpiperidine-1-oxyl (TEMPO) was used to detect photogenerated holes and electrons. The presence of superoxide and hydroxyl radicals was determined using 5,5-dimethyl-1-pyrroline *N*-oxide (DMPO) in dimethyl sulfoxide and water, respectively.

### 2.3 Antibacterial Activity

#### 2.3.1 Growth-Based Viability Method in Studying the Antibacterial Activity under Dark Conditions

Colonies of *E. coli* K-12 were cultured by streaking a frozen glycerol stock onto LB agar plates, followed by incubation at 37 °C for 24 h. A single colony was then transferred to Tryptic Soy Broth (TSB) to prepare a bacterial stock suspension, which was grown to the stationary phase overnight at 37 °C with continuous shaking at 150 rpm. Before each experiment, the bacterial cultures were washed twice with sterilized saline. A growth-based viability assay modified from the method by Qiu et al. was used to evaluate bacterial viability.^30^ Serial dilutions (1:1) of carbon suspended in sterilized saline were prepared in an exposure plate (96-well plate). Bacteria were then added to each well to achieve a final concentration of 7 log colony-forming units (CFU)/mL. Three columns of each 96-well plate, containing bacteria without carbon particles, were used to construct a viability calibration curve. In these columns, the first well received 7 log CFU/mL (defined as 100% viability), followed by serial dilutions down subsequent wells. An additional column containing only media served as a sterility control. The exposure plate was shaken at 150 rpm at room temperature and subsequently incubated at 37 °C for 1 h without shaking. After incubation, 5 μL from each well was transferred to 195 μL of fresh TSB growth medium in a new 96-well plate. Bacterial growth was monitored by measuring the optical density at 600 nm (OD600) over 16 h at 37 °C using a plate reader (SpectraMax Mini, Molecular Devices). Growth curves were analyzed using R code published by Qiu et al. to quantify bacterial viability.^30^

#### 2.3.2 Colony Counting Method in Studying the Antibacterial Activity under Dark and Visible Light Conditions

Colonies of *E. coli* K-12 were cultured by streaking a frozen glycerol stock onto LB agar plates, followed by incubation at 37 °C for 24 h. A single colony was then transferred into Tryptic Soy Broth (TSB) to prepare a bacterial stock suspension, which was grown to the stationary phase overnight at 37 °C with continuous shaking at 150 rpm. Before each experiment, the bacterial cultures were washed twice with sterilized saline. Then, the culture was serially diluted with sterilized saline. As a source of visible light, a solar light simulator (Newport LCS-100) excited by a 100 W Xeon lamp with an AM1.5G filter, a UV cutoff filter (>400 nm), and an infrared radiation cutoff filter (Newport KB-2, <700 nm) was applied. The spectra of the light source applied to the carbon samples are shown in Figure S1. The specific light intensity exposed to the samples was 181 (±2) mW/cm^2^. The light was irradiated from below with the well containing 4 mg/mL carbon particles with 7 log CFU/mL *E. coli*. They are mixed at 150 rpm in the dark and then exposed to light without shaking. In different time intervals, 100 μL or 10 μL of mixed solutions was serially diluted and dropped onto Luria–Bertani (LB) agar plates. It is worth mentioning that in order to obtain precise results, the entire solution (200 μL) was introduced to the plates under the condition of C-2 visible light for 1 h. The plates were incubated overnight at 37 °C, and the colonies were counted the following day to confirm the concentration of viable cells. Experiments conducted under dark conditions were performed on a 96-well plate wrapped with aluminum foil to ensure darkness. All experiments were carried out in triplicate, and the average and standard deviation values were calculated for analysis.

The antibacterial effect of C-2 (4 mg/mL) was evaluated against Gram-positive *Staphylococcus epidermidis* (*S. epidermidis*, ATCC 14990, initial concentration of 6 log CFU/mL) under both dark and visible-light conditions for 1 h.

#### 2.3.3 Cycling Test

Reusability tests were performed using 4 mg/mL of C-2. Following each antibacterial cycle, C-2 was sterilized with ethanol, dried overnight, and reused against *E. coli* (7 log CFU/mL). The bacterial concentration of the supernatant (100 μL) was determined by a colony counting method.

#### 2.3.4 Sulfur Leaching Test

The leachate of C-2 (4 mg/mL) in Milli-Q water under visible light and dark conditions for 1 h was analyzed using an inductively coupled plasma mass spectrometer (ICP-MS, Thermo Fisher Scientific, Bremen, Germany) and ion chromatography (IC, Thermo Scientific Dionex Aquion). The ICP-MS and IC were used to quantify total sulfur and sulfate concentrations, respectively. The calibration curves for quantifying them are shown in Figure S2.

#### 2.3.5 Fluorescent-Based Cell Live/Dead Test

The bacteria’s death analysis was also ascertained by the fluorescence-based cell live/dead test at 1 h. The suspension was stained with propidium iodide (PI) and SYTO9 using the LIVE/DEAD BacLight Bacterial Viability Kit, following the manufacturer’s instructions, and subsequently imaged with a laser scanning fluorescence microscope (Zeiss AXIO Imager). The images were acquired with an Axiocam 503 color camera and ZEN LITE software. The mounted specimens were observed using a 20× lens. The cell suspension exposed to visible light in the absence of C-2 served as the control group.

#### 2.3.6 Reactive Oxygen Species Detection-Scavenger Experiment

To investigate whether different reactive species play a role in the inactivation of *E. coli*, specific radical scavengers were added to the medium to selectively suppress the activity of targeted species under visible light irradiation for 1 h. Specifically, 0.5 mM sodium oxalate was used to eliminate the influence of holes, 0.05 mM Cr­(VI) was used as an electron scavenger, and 0.5 mM isopropanol (IPA) was added to remove hydroxyl radicals (•OH). The colony-counting method was used to quantify the concentration of *E. coli* in this experiment.

#### 2.3.7 Toxicity Evaluation

HFOB cells were used for testing toxicity. A commercially available micromeso porous carbon BAX (Nuchar BAX 1500) was used as a control since it has a similar surface area as C-1. First, C-1, C-2, and BAX carbon samples were dispersed in cell culture medium at a concentration of 0.5 mg/mL and sonicated for 20 min to ensure uniformity. HFOB cells were seeded into a 24-well plate at a density of 20,000 cells per well. One day after seeding, the medium containing the different carbon samples was added. Cell proliferation was recorded on days 0, 1, and 3. Cell culture was performed using DMEM/F-12 cell culture medium supplemented with 10% FBS (fetal bovine serum) and 1% Pen/Strep, cultured at 37 °C in a 5% CO_2_ incubator. Cell proliferation was assessed by using the CellTiter-Blue Cell Proliferation Kit.

## 3 Results and Discussion

### 3.1 Surface Chemistry and Structure Characterizations

FT-IR spectra of carbons are shown in Figure 1a. The spectrum for C-2 exhibited more intense bands than that for C-1, which is attributed to its lower degree of aromatization of this carbon. C-1 showed fewer peaks due to the loss of functional groups during carbonization at a higher temperature. The vibrations corresponding to C–H bonds in methyl groups appeared at 3020 cm^–1^.^31^ Stretching vibrations associated with C=O groups (such as those in carboxylic acids, ketones, lactones, and aldehydes) are typically observed around 1720 cm^–1^.^31^ Stretching vibrations of C=C bonds in aromatic rings appear near 1560 cm^–1^. On the spectrum of the C-1 carbon, a band at 600 cm^–1^ is visible, indicating the bending vibrations in its aromatic structure. For C-2 carbon, the vibration bands at 1029 cm^–1^ and 1140 cm^–1^ are assigned to the symmetric and asymmetric O=S=O stretching modes of the sulfonic group (SO_3_H), respectively.^32^ Bands in the 700–900 cm^–1^ region are likely associated with polyaromatic structures and related functional groups.^33^ A broad band centered around 1186 cm^– 1^ is attributed to C–O–C or O–S vibrational modes.^34^

**Figure 1:**
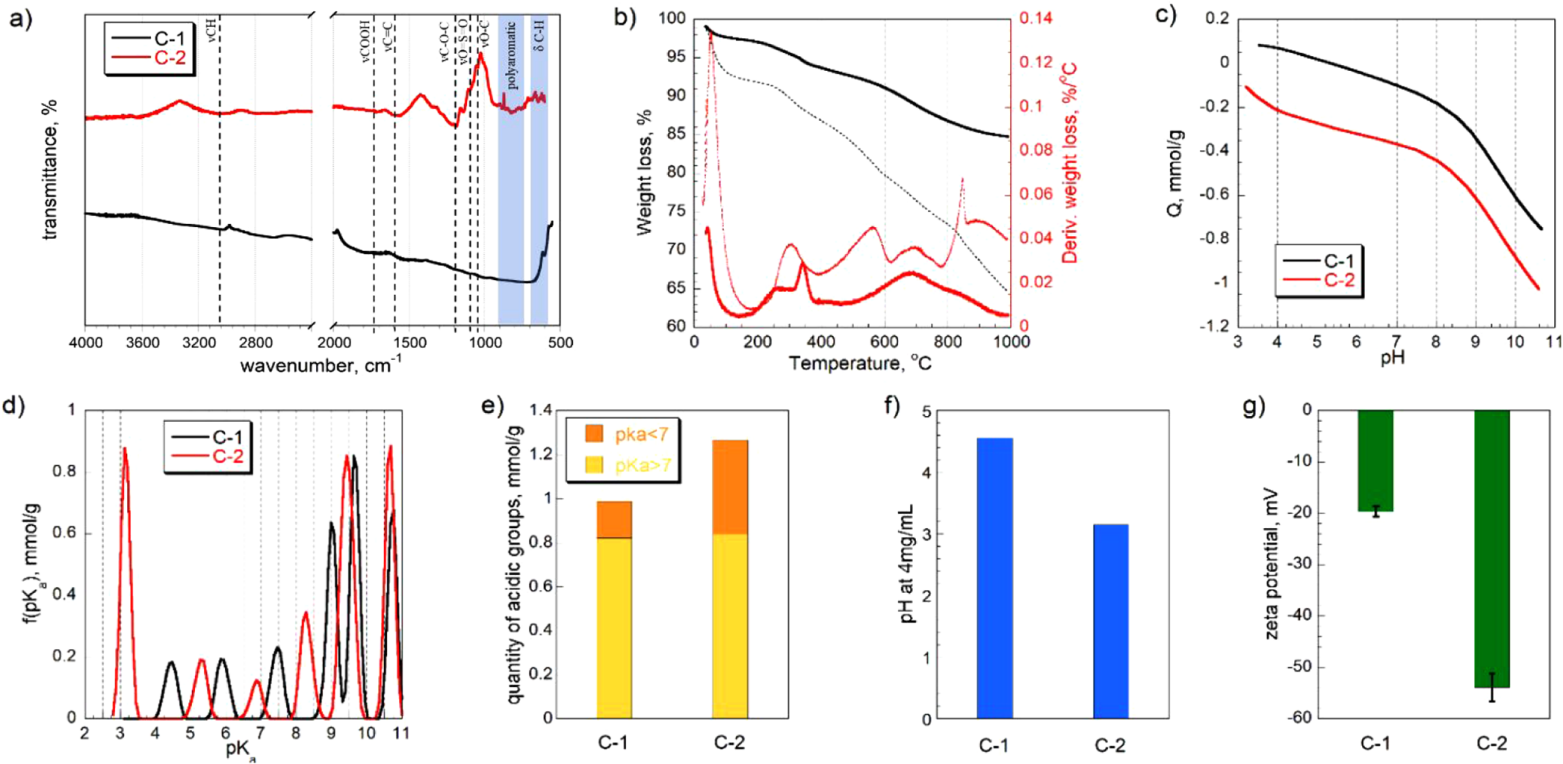
a) FT-IR spectra, b) TGA and DTG curves in N_2_ atmosphere (solid line represents C-1 and dotted line represents C-2), c) proton binding curves, d) p*K*_a_ distributions, e) the amounts of surface acidic groups categorized based on their p*K*_a_ value, f) pH at 4 mg/mL in saline, and g) zeta potential of C-1 and C-2.

Thermogravimetric analysis (TGA) results (Figure 1b) showed that the thermal decomposition of groups on sulfur-doped carbons occurred in three stages. The peak at 100 °C on the derivative thermogravimetric (DTG) curves is linked to the release of water. The oxygen-containing species, such as carboxylic groups and sulfonic acids, decompose between 200 and 400 °C.^35,36^ The lactones/sulfoxides/sulfones groups are released at 580 °C.^35^ At 710 °C, phenolic groups decomposed.^37^

The proton binding curves shown in Figure 1c indicate the acid–base nature of the carbon tested. The negative values of proton uptake show that the materials release protons.^28^ C-2 exhibits acidic character, whereas C-1 displays a smaller contribution from acidic surface groups. The surface pH of C-1 and C-2 was 5.16 and 3.55, respectively, which indicates that polymer-derived carbons carbonized at lower temperatures tend to retain more acidic surface functional groups than carbons treated at higher temperatures. The results are consistent with those from TGA showing that the carbon carbonized at a lower temperature retains more oxygen-containing species. The total amount of acidic groups (Figure 1e) with different p*K*_a_s on C-2 is higher than that on C-1 (1.266 mmol/g vs 0.966 mmol/g). The peaks on the distributions (Figure 1d) correspond to acidic groups of different p*K*_a_s representing various types of oxygen and/or sulfur heteroatoms in the functional groups. 17% of sites (0.167 mmol/g) on C-1 and 38% (0.429 mmol/g) on C-2 are strongly acidic groups with large acidic dissociation constants, likely carboxylic groups (p*K*_a_ = 3–7).^38^ Peaks with higher p*K*_a_s (9–11) on both carbons are linked to phenolic groups and thiophenol groups^39^ A zeta potential (Figure 1g) indicates that the surface charge of both carbons is negative due to the deprotonation behavior of acidic surface functional groups.

XPS analysis was performed to investigate the surface chemical environment in detail. Both polymer-derived carbons contain not only carbon (C) but also oxygen (O) and sulfur (S) elements. The elemental composition in atomic percent and the deconvolution of the C *1s*, O *1s*, and S *2p* core energy level spectra are shown in Table 1 and Figure 2. The surface sulfur content was 1.5% for C-1 and 4.1% for C-2. As the carbonization temperature increased, the carbon content also increased. C-1 showed a higher amount of C–O and O–C=O compared to C-2. From the deconvolution of the O *1s* core energy level spectra, more oxidized carbon species are present on C-2 carbon than on C-1. The S *2p* deconvolution showed that C-2 possesses a higher amount of sulfur-containing species and, in each category, than does C-1. Besides, the sulfonic acid groups contribute significantly to its acidity, which supports the low surface pH of C-2. They fall outside the detection limit of potentiometric titration. In summary, XPS reveals detailed information that C-1 possesses higher amounts of C–O, C=O, and O–C=O species, while C-2 contains higher amounts of sulfur-containing species.

**Table 1:** Deconvolution Results of the C *1s*, O *1s,* and S *2p* Core Energy Level Spectra for the Carbons (the Surface Amounts Are in at. %)

Bonding energy, eV		**C-1**	**C-2**
**C** ***1s***		89.0	81.0
284.8	C=C (sp^2^, graphitic)	62.2	67.4
286.6–286.8	C–O (phenolic, alcoholic, etheric), C–S (carbon–sulfur structures)	12.5	6.3
288.2–288.3	O=C/O=S (in carbonyl/carboxyl or sulfoxides/sulfones)	7.5	2.6
289.6–289.8	O–C=O (carboxyl or ester)	4.3	3.3
291.2	π–π* electrons in aromatic ring	2.4	1.4
**O** ***1s***		9.5	14.8
531.5–532.1	O=C/O=S (in carbonyl/carboxyl or sulfoxides/sulfones)	5.8	10.1
533.3–533.6	O–C/O–S (in phenol/epoxy or thioethers/sulfonic)	3.7	4.7
**S *2p*** ***3/2***		1.5	4.1
163.4–164.2	thiophenic/sulfide configurations	0.88	2.39
168.1–168.5	R-SO_2_-R (in sulfoxides/sulfones)	0.39	1.16
169.7	R-SO_3_H (in sulfonic)	0.20	0.58

**Figure 2:**
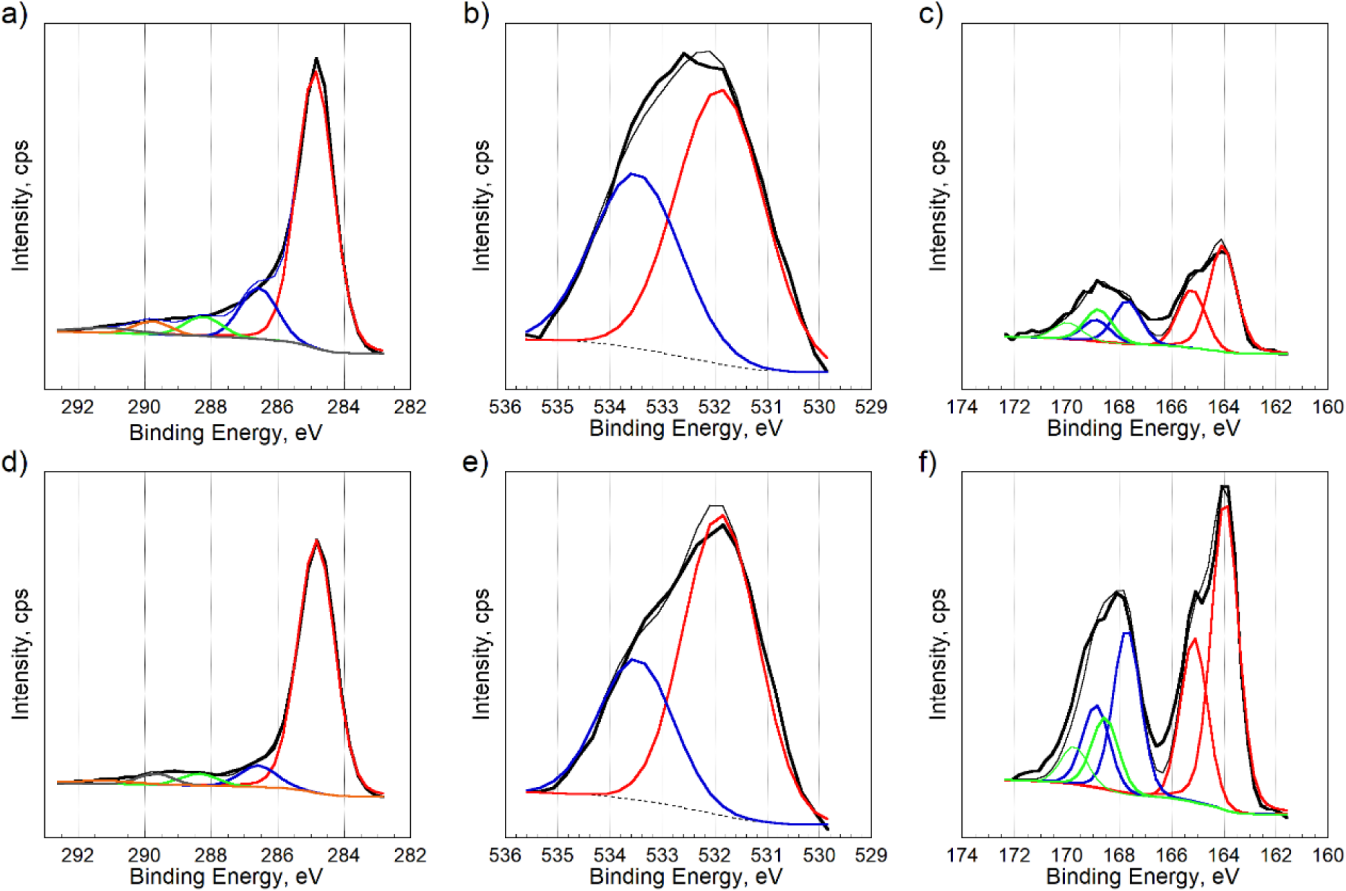
C *1s*, O *1s,* and S *2p* of C-1 (a–c) and C-2 (d–f).

The porosity of carbons was analyzed by nitrogen adsorption. The BET surface area of C-1 and C-2 is 1322 m^2^/g and 364 m^2^/g, respectively. The carbonization temperature has an important effect on carbon porosity (Figure 3). The pore size distribution demonstrates the presence of meso- and micropores. As the pyrolysis temperature increased, the porous structure developed, especially the micropores (Figure 3c) C-1 showed a surface area 3.6 times higher than that of C-2. *E. coli* have an average surface area of 6.7 μm^2^ per cell, with a mean size of approximately 2 μm;^40^ only pores larger than 2 μm are accessible to these bacteria. Based on the pore size distribution of sulfur-doped carbon, *E. coli* cannot enter its pores. Nevertheless, bacteria can still adhere to the carbon particles. TEM images (Figure S3) further revealed structural differences between C-1 and C-2. C-1 exhibited surface pores larger than those of C-2.

**Figure 3:**
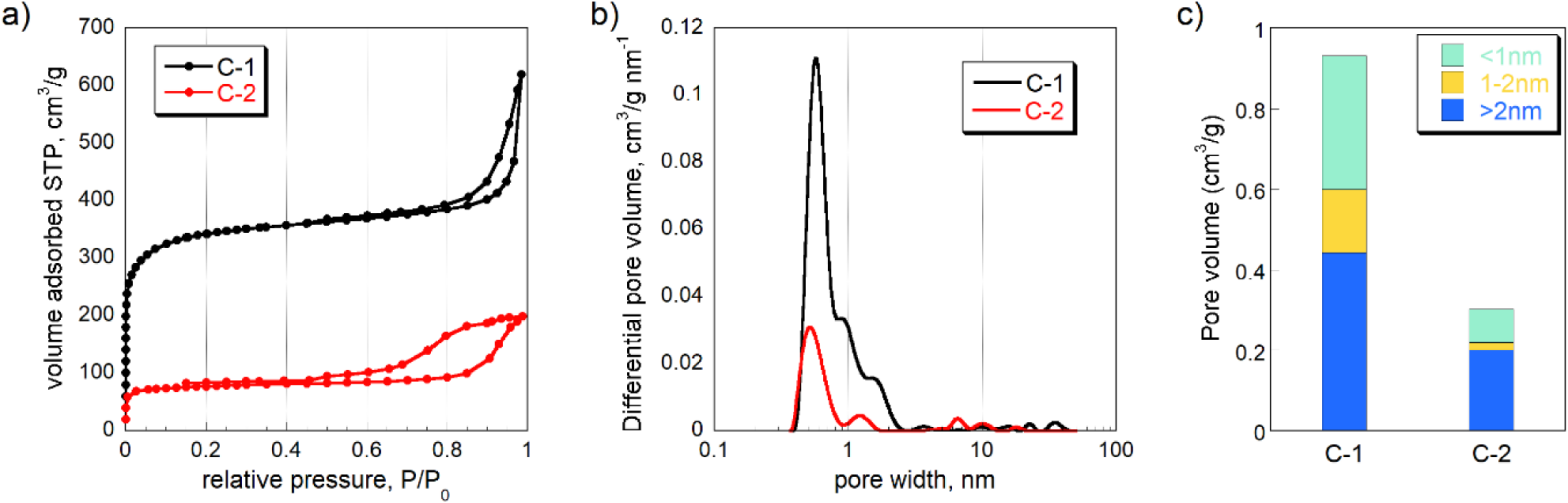
a) Nitrogen adsorption isotherms at −196 °C, b) pore size distributions, and c) pore volume distributions of C-1 and C-2.

### 3.2 Antimicrobial Activity of Carbon under Dark and Visible Light Conditions

The antibacterial activity under dark conditions was evaluated by a growth-based viability assay. *E. coli* (7 log CFU/mL) was incubated with different doses of carbons for 1 h in a saline solution. The results (Figure 4a) showed a dosage-dependent antibacterial effect for the C-2 carbon but not for the C-1 carbon. Bacteria usually have a net negative charge, primarily due to the presence of groups such as carboxyl, amino, and phosphate on the cell wall. The role of surface charge in bacterial adhesion remains ambiguous, as various studies have produced conflicting results.^41^ While many studies have indicated that negatively charged surfaces tend to inhibit bacterial attachment, others have shown that bacteria can overcome electrostatic repulsion and adhere strongly to negatively charged materials, often through the aid of their surface appendages. In our case, both carbons showed a negative zeta potential (Figure 1g) in the saline solution. The microscopic (Figure S4) and TEM images (Figure 5a,b,d,e) showed that some *E. coli* cells were attached to these carbon surfaces, while small carbon particles were also observed adhering to the *E. coli* cell walls. The concentration of *E. coli* (7 log) in the solution did not show a noticeable decrease in the presence of C-1 carbon during the test, suggesting that the initial concentration of *E. coli* was much higher than the amount absorbed onto the carbon surface. C-2 has a lower surface area than that of C-1, and the number of *E. coli* attached to C-2 is also negligible under this concentration. Therefore, quantifying the number of *E. coli* in the solution is not affected by the number of *E. coli* attached to their surface. It is worth mentioning that the suspension was not shaken during the antimicrobial test; therefore, the killing effect is less likely to be linked to any physical damage.

**Figure 4:**
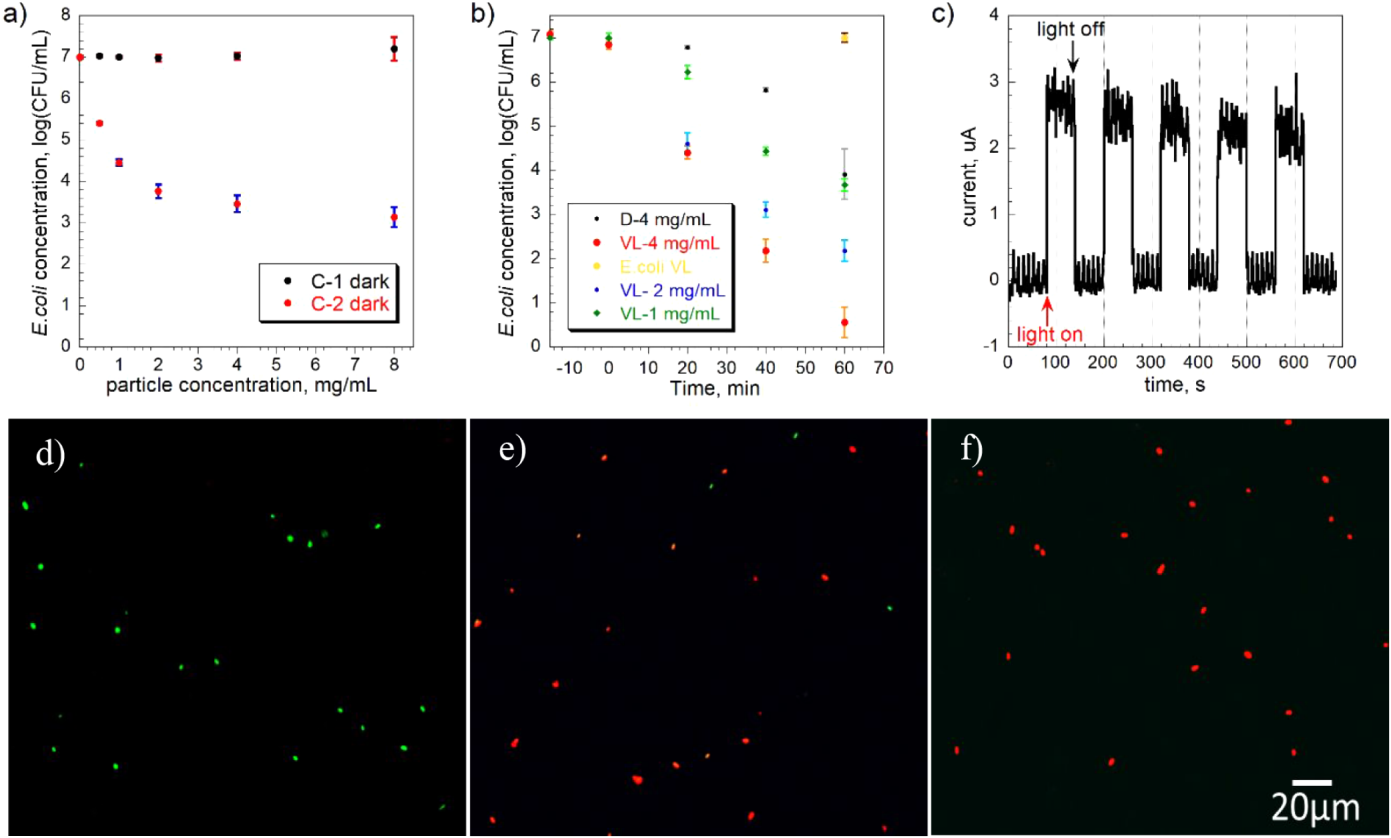
a) Effect of C-1 and C-2 on the *E. coli* concentration measured by the growth-based viability assay test under dark conditions for 1 h in isotonic saline, b) effect of C-2 on the *E. coli* concentration under various conditions assessed by the colony counting method (cultured *E. coli* cells treated under visible light in the isotonic saline are named as *E. coli* VL (control visible light)), c) transient photocurrent of C-2 upon visible light; enlarged fluorescence images of live (green) and dead (red) cells of d) control visible light, e) dark condition with C-2, and f) visible light condition with C-2. Scale bar: 20 μm.

**Figure 5:**
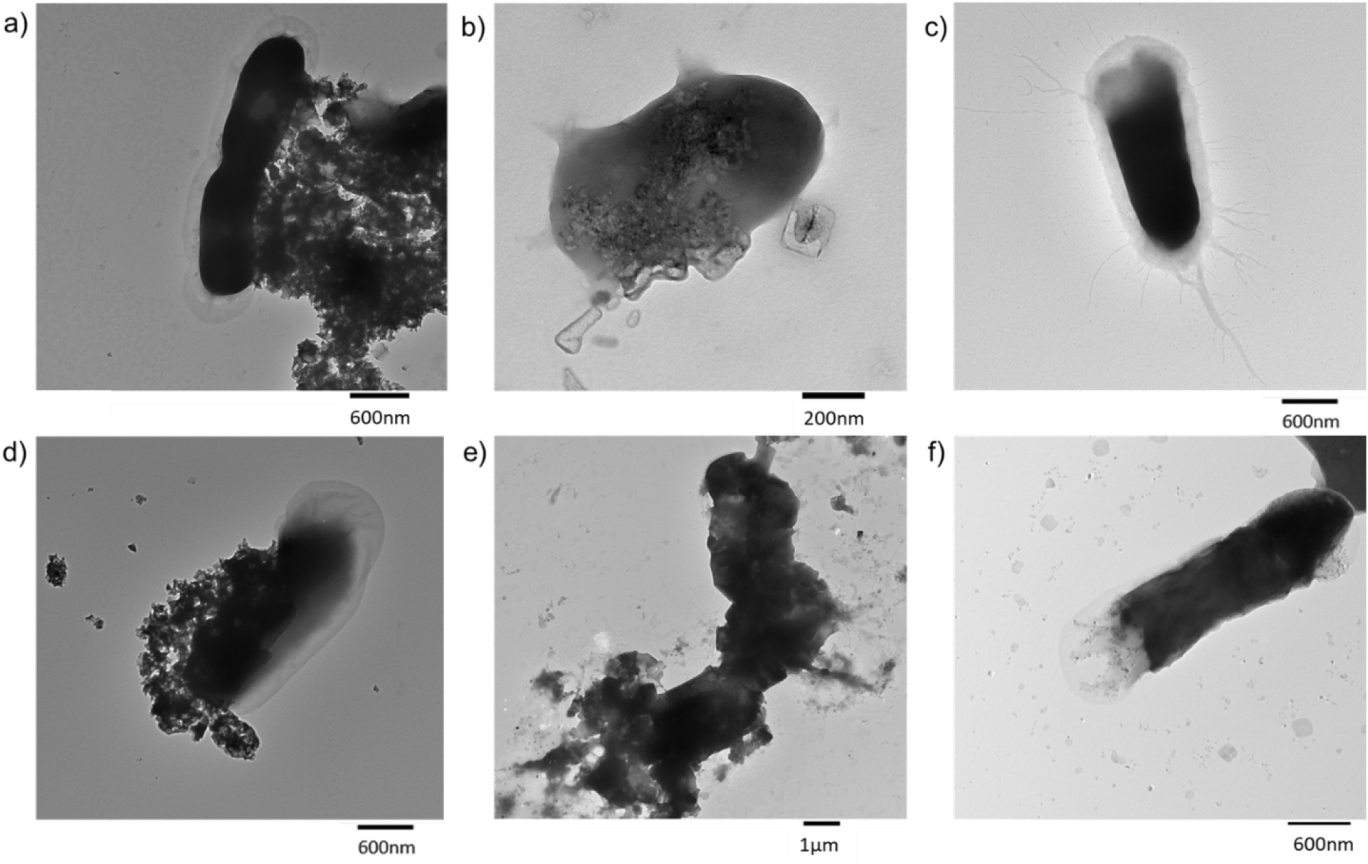
TEM image of *E. coli* under a) C-1 visible light, b) C-2 visible light, c) control visible light, d) C-1 dark, e) C-2 dark, and f) control dark conditions for 1 h.

At dosages of 2 and 4 mg/mL, C-2 carbon exhibited 3-log and 4-log reduction, respectively, in *E. coli*, which indicates it effectively inhibits bacterial survival under dark conditions. A lower external pH of 4.4 is known to lower the intracellular pH to approximately 6.0 and adversely affect cellular macromolecules. The resulting acid stress leads to decreased enzyme activity, acid-induced protein unfolding, membrane damage, and DNA damage.^42^ Linking the surface chemistry characteristics shown in Figure 1, the decrease in the bacterial population is due to the high release of protons from the C-2 carbon. To further verify the extent of the proton effect, pH control experiments were conducted by exposing *E. coli* to saline solutions at different pH values under dark conditions. At pH 3.50 and 3.00, the concentrations showed 2.6-log and 3.5-log reduction, respectively, in *E. coli* after 1 h. These results confirm that acidic conditions alone can inhibit bacterial viability.

It is therefore plausible to assume that surface chemistry, especially acidic groups such as sulfonic acid and carboxylic acids on the surface of C-2, plays a major role in its bactericidal effect in an aqueous environment. XPS analysis revealed that C-2 possesses a more oxidized surface than C-1 and contains a higher amount of sulfonic acid groups, which contribute to its enhanced surface acidity. Even though C-1 has a more developed porous structure than C-2, it does not exhibit antibacterial activity. This suggests that surface porosity alone, in the absence of high surface acidity, does not contribute to bacterial entrapment or inactivation. The pore sizes of C-1 are likely too small to effectively retain *E. coli*, emphasizing that surface chemistry rather than physical texture is the dominant factor governing the antibacterial performance of these carbons.

As the next step, the effect of visible light on the antibacterial activity of C-1 and C-2 was studied. One hour of light exposure alone had a negligible effect on *E. coli* viability (Figure 4b). When tested at different concentrations, C-2 exhibited a dosage-dependent antibacterial effect under visible light. With an increment of irradiation time from 20 to 60 min, C-2 showed a time-dependent antibacterial effect under both dark and illuminated conditions. Under dark conditions, bacterial viability progressively decreased with longer exposure times. A 3–4 log reduction was observed after 1 h. The reduction was more dramatic under visible light, reaching 6–7 logs within the same period. To validate these results, a fluorescence-based live–dead assay was conducted. The assay kit contains two nucleic acid-binding stains: SYTO 9 and propidium iodide. SYTO 9 penetrates all bacterial membranes, staining the cells green. While propidium iodide penetrates only cells with damaged membranes, the combination of the two stains results in red-fluorescing cells. As expected, almost all cells were observed with green fluorescence when they were exposed just to light alone (Figures 4d and S5a). After 1 h under dark conditions (Figures 4e and S5b), several green fluorescent cells were observed. The majority of red fluorescent cells indicates that most of the bacteria were dead or their membranes were largely damaged. Under visible light (VL) (Figures 4f and S5c), almost all cells exhibited red fluorescence. C-1 did not show a noticeable antibacterial effect under visible light for 1 h. The effect of C-2 on the Gram-negative *S. epidermidis* concentration was assessed (Figure S7). The log reduction under dark and visible light conditions was 0.8 and 1.4, respectively. It is important to note that the colony counting method has a 10% error,^43^ especially when successive serial dilutions are needed to achieve countable results. Thus, a precise quantification of the differences in bacterial populations in contact with C-1 under dark and visible light conditions became challenging when the *E. coli* concentration in both cases was approximately 7 log CFU/mL. As for C-2, the entire suspension of sample C-2, after 1 h of visible light exposure, was applied directly to the agar plate without any serial dilution (Figure S6), which made it possible to quantify the number of bacteria with more precision.

The interactions between the carbons and *E. coli* are illustrated in the TEM images (Figure 5), which show *E. coli* cells after exposure to C-1 and C-2 under dark and visible light conditions, along with the corresponding controls. These images clearly reveal that the edges of the carbon particles are in direct contact with the bacterial cells. The observed interaction sites are similar to those previously reported for reduced graphene oxide^3^ and graphitic carbon nitride.^44^ Fimbriae were clearly seen in controls, which are displayed as gray halos around the bacterial cells (Figure 5c,f).^45,46^ The gray halos were not observed in *E. coli* treated with C-2, whereas they were clearly visible in the *E. coli* treated with C-1. For the C-2-treated *E. coli* under both dark and light conditions, the disappearance of fimbriae may indicate that the bacterial cells are under environmental stress, potentially caused by pH, osmolarity, or oxidative stress.^47,48^ The surface of the cell wall also became roughened (Figure 5e) under dark conditions, indicating signs of membrane damage. The cell wall was seen to be damaged under visible light, and its loss led to bacterial lysis (Figure 5b).

Control experiments with light exposure alone confirmed that bacterial death could not be attributed to photothermal effects. Notably, 1 h of visible light exposure did not significantly raise the temperature of the carbon-treated bacterial suspensions; the temperature reached only 35 °C after 1 h (Figure S8), further excluding the contribution of photothermal effects. The pH of the C-2 suspension remained the same under dark and visible light exposure (pH = 3.15).

### 3.3 Cytotoxicity of Sulfur-Doped Carbons

To investigate whether sulfur-doped porous carbon materials are safe for use in antimicrobial applications without harming host cells, it is necessary to test the cellular compatibility of the carbon samples. As depicted in Figure 6, the cell proliferation profiles of the C-1 and C-2 experimental groups remained consistently comparable to the control group (no carbon) throughout the 3-day culture period. Statistical analysis revealed no significant differences in proliferation rates, indicating that neither C-1 nor C-2 elicited cytotoxic responses in the hFOB cells. These findings support the conclusion that both carbon materials exhibit high cellular compatibility. It is important to note that the commercially available sulfur-free carbon sample BAX (Nuchar BAX 1500) was also included for comparison with C-1 and C-2. BAX demonstrated a markedly enhanced pro-proliferative effect relative to the control group. This observation is likely attributable to BAX’s physicochemical characteristics, including its high surface area (*S*_BET_ = 1541 m^2^/g) and neutral pH (7.42), which collectively promote osteoblast adhesion and proliferation.

**Figure 6:**
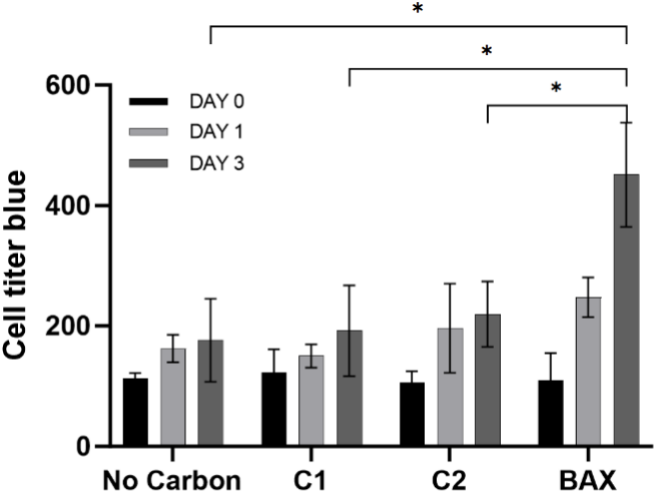
Cell toxicity test of HFOB cells assessed by the CellTiter-Blue kit at day 0, day 1, and day 3 (*p < 0.05, *n* = 4).

### 3.4 Proposed Mechanism of Disinfection by Photoactive S-Containing Carbon

Light-induced bacterial inactivation is commonly ascribed to oxidative stress caused by the generation of reactive oxygen species (•O^2–^, •OH, H_2_O_2_, and ^1^O_2_), along with contributions from photogenerated holes (h^+^) and electrons (e^–^). Photocurrent response and Mott–Schottky measurements were used to assess electron–hole separation and energy bandgap characteristics. As shown in Figure 4c, C-2 showed an increase in photocurrent under intermittent light irradiation, indicating efficient separation of photogenerated charge carriers.^27,49^ The Mott–Schottky plot (Figure S9) for C-2 shows both n-type and p-type semiconductor features,^50^ which indicate the coexistence of both free electrons and holes. The estimated band gap of C-2 is 1.8 eV based on the flat potential of the conduction band and valence band, which supports the phenomenon of anodic photocurrent generation upon visible light. The semiconducting behavior correlates with the surface chemistry characteristics; electron-withdrawing groups such as sp^3^-bonded carbonyl, carboxyl, and hydroxyl groups would likely induce p-type conductivity.^51^ Sulfur species such as sulfone, sulfoxide, and sulfonic groups (−SO_3_H) also act as p-type dopants due to their strong electron-withdrawing nature.^52^ In contrast, electron-donating groups such as sp^2^-bonded hydroxyl, ether, and epoxide groups induce n-type conductivity.^51^

To identify which reactive species play an important role in the bactericidal activity of C-2 under visible light, EPR analysis was performed. The signal intensity of TEMPO (2,2,6,6-tetramethylpiperidine 1-oxyl) decreased upon exposure to C-2 under light, which indicates that both electrons and holes were effectively captured by TEMPO (Figure S10). However, the hydroxyl radical, superoxide, and singlet oxygen were not detected under the conditions of 5,5-dimethyl-1-pyrroline *N*-oxide (DMPO) in DMSO, DMPO in water, and 2,2,6,6-tetramethylpiperidine (TEMP), respectively. This indicates that the generated electrons did not participate in the formation of these reactive oxygen species. To further investigate the photocatalytic bacteria disinfection mechanism, a series of scavenger experiments was conducted by adding individual scavengers to the photocatalytic reaction system containing bacteria. In the experiment, 0.05 mM sodium oxalate, 0.05 mM Cr­(VI), and 0.5 mM isopropanol were used to capture holes, electrons, and hydroxyl radicals, respectively. These scavengers have been widely used to study the photocatalytic mechanism both in pollutant decomposition^53^ and bacterial inactivation.^54–57^ It is important to mention that none of the scavengers imposed a toxic effect on *E. coli* at this level of concentration.^56^ As shown in Figure 7, the addition of Cr (VI) or isopropanol to C-2 resulted in minimal changes in the disinfection efficiency under visible light, whereas the presence of sodium oxalate significantly suppressed the antibacterial activity compared with other scavengers. The results under dark conditions also show that the pH changes due to the added scavengers had a negligible influence on the disinfection efficiency. With the addition of 0.05 mM sodium oxalate, the number of *E. coli* became similar to that under dark conditions. These results indicate that the photocatalytic disinfection of C-2 is primarily driven by a hole-induced oxidation-dominated process under visible light radiation. The generated holes likely exert their bactericidal effect on cells that are attached to the carbon surface.^58^

**Figure 7:**
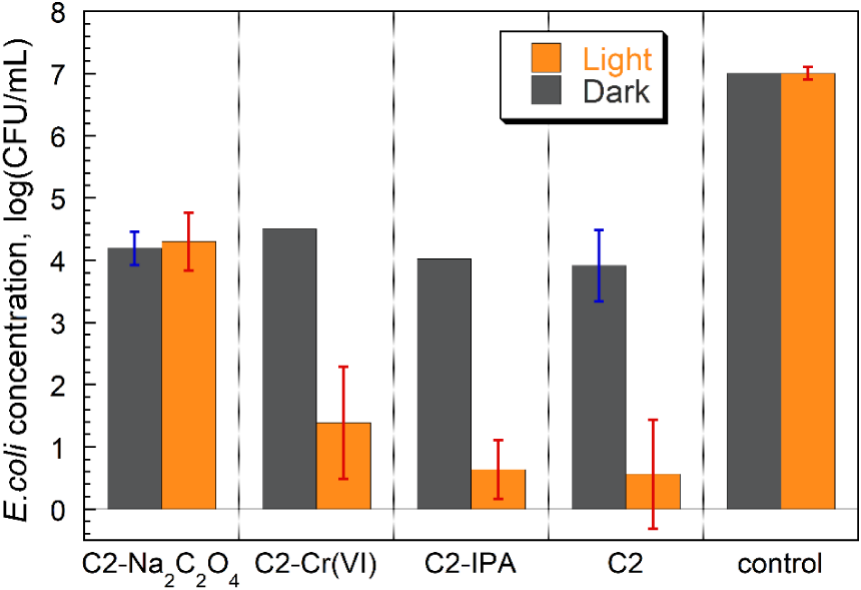
Photocatalytic disinfection efficiency against *E. coli* (7 log CFU/mL) in the presence of C-2 (4 mg/mL) with different scavengers (0.5 mmol/L sodium oxalate, 0.05 mmol/L Cr (VI), and 0.5 mmol/L isopropanol) under dark conditions, visible light conditions, and control (without C-2) conditions.

Gram-negative *E. coli* is more susceptible to C-2 than Gram-positive *S. epidermidis*. This difference in susceptibility is likely due to the distinct structural features of the cell envelopes. In *E. coli*, the outer membrane contains lipids and proteins that are vulnerable to oxidative damage by the generated holes. *S. epidermidis* lacks an outer membrane and instead possesses a thick peptidoglycan layer that provides additional protection. The thick, rigid cell wall and stable peptidoglycan structure of *S. epidermidis* contribute to its high resistance to C-2. Similar findings have been reported when the killing mechanism involves oxidation or the generation of reactive oxygen species.^59,60^

Other sulfur-doped carbons, when exposed to visible light, were also reported to be active in hole formation^61^ or photocurrent generation.^24,27,53,61^ In the work by Xia’s group,^61^ lignosulfonate-derived sulfur-doped carbons were applied for the photocatalytic degradation of tetracycline under visible light. Transient photocurrent response was detected upon light, and EPR confirmed the generation of superoxide, hydroxyl radicals, and holes. In our work, the number of *E. coli* killed by the holes generated from C-2 is about 2500 CFU/mg, which is significantly smaller than the effect of protons dissociated from the carbon (∼2.49 × 10^6^ CFU/mg). The protons play a dominant role in the observed bactericidal effect. This conclusion is also supported by the fluorescent image of C-2 under dark conditions (Figure 4e and S2b), which shows that the majority of the bacteria are killed, likely due to the proton dissociation from C-2.

To evaluate the reusability of C-2, a cycling test was performed at 4 mg/mL. As shown in Figure 8, the antibacterial efficiency showed a 5.3 and 3.4 log reduction at the second and third cycles, respectively. The possible reason for the reduced bacterial inhibition may be that the debris of bacteria was adsorbed onto the carbon surface. The loss of sites that generate holes in the course of the cyclic experiments may be the other reason. To further investigate the cause of the decreased antibacterial performance and the stability of the carbon material under light was analyzed for possible leaching of sulfur species. The C-2 suspension in water was analyzed using ICP-MS under 1 h of dark and visible light conditions. Sulfur was detected under dark conditions (25 ± 2 ppm) due to leaching from the carbon itself, which comes from the carbonization of the polymer, poly­(sodium 4-styrenesulfonate), even though the carbon was intensively washed after carbonization. We then performed ion chromatography on the same leachates, confirming that all of the sulfur was present in sulfate form. After visible light, there was 26 ± 2 ppm sulfate in the leachate. The increment of sulfate can be attributed to the oxidation of the sulfonic acid group, where the C–S bond breaks and is released as sulfate.

**Figure 8:**
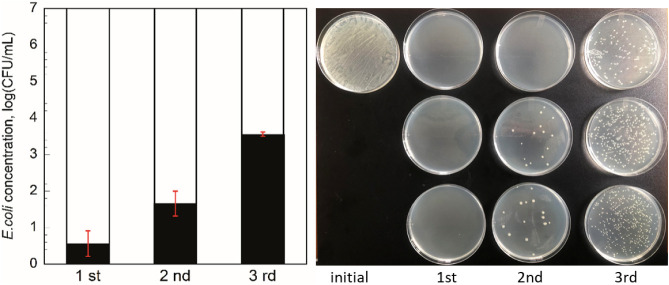
Cyclic disinfection of C-2 (3 cycles) with agar plate images.

Graphene oxide and graphitic carbon nitride (GCN) have been reported in the literature as metal-free disinfection materials (Table 2) under visible light.^16,62–66^ This study presents a novel investigation into the disinfection activity of sulfur-doped porous carbons, which has received limited attention in antimicrobial applications. Notably, acidic groups, particularly sulfonic acid moieties, appear to govern the antibacterial activity under dark conditions. This antibacterial effect is likely mediated by the release of protons. Under visible light irradiation, the role of sulfur functionalities becomes more pronounced. Sulfur species such as sulfones, sulfoxides, and sulfonic groups are known to act as strong electron-withdrawing dopants, which affect the electronic structure of the carbon matrix. These groups facilitate the generation of photogenerated holes, thereby promoting oxidative stress in microbial cells.^67^ The antibacterial properties of sulfur-doped porous carbon may offer a specific advantage that surpasses those of graphene-based or graphitic carbon nitride-based materials. Because this study represents the first demonstration of sulfur-doped carbon as a photocatalyst for disinfection, the focus was placed on establishing synthetic feasibility and antibacterial performance rather than quantitative quantum yield determination. Future studies could focus on optimizing sulfur speciation and evaluating the quantum yield of the material. In addition, single-wavelength experiments could be performed to clarify the wavelength-dependent behavior and provide deeper insight into the hole formation mechanism.

**Table 2:** Comparison of the Antibacterial Activity of Metal-Free Materials Reported in the Literature

Material	Target microorganism/concentration	Particle concentration	Inactivation time (min)	Reduction under dark	Reduction Under light	Light source	Ref.
Graphene Oxide	*E. coli* K-12/1E6–1E7	25 μg/mL	30	0.16 log (31.2%)	0.6 log (75.1%)	450 W Xe lamp with k\1.5G solar simulation	^16^
CNRGOS_8_ RGOCNS_8_	*E. coli* K-12/2E6	Not indicated	240	Negligible	6.3 log (100%)	300 W Xe lamp with k\400 nm filter	^62^
g-C_3_N_4_	*E. coli* K-12/2.5E6	1 mg/mL	240	Negligible	6.4 log (100%)	300 W Xe lamp with k\400 nm filter	^54^
Porous g-C_3_N_4_ (PCNS)	*E. coli*/5E6	0.4 mg/mL	240	Negligible	6.7 log (100%)	500 W Xe lamp with k\420 nm filter	^63^
Graphene oxide/g-C_3_N_4_	*E. coli*/1E7	0.1 mg/mL	120	Negligible	1.7 log (97.9%)	300 W Xe lamp with k\420 nm filter	^64^
Sulfur-doped GCN	*E. coli*/1E7-1E8	2 mg/mL	100	Not indicated	7–8 log (100%)	300 W Xe lamp with k\420 nm filter	^65^
This work	*E. coli* K-12/1E7	4 mg/mL	60	3–4 log	6–7 log	100 W Xe lamp with k\1, 0.5Gk\400 nm filter and k\IR filter	

## 4 Conclusions

Metal-free sulfur-doped porous carbon on an acidic surface demonstrated high bactericidal activity. Under dark conditions, exposure of *E. coli* to the sulfur-doped carbon for 1 h led to a 3–4 log decrease in bacterial concentration from an initial concentration of 7 log CFU/mL. When exposed to visible light, the antibacterial effect was enhanced, achieving a 6–7 log decrease. To the best of our knowledge, this is the first comprehensive study of the antibacterial activity and mechanism of sulfur-doped carbon under dark and visible light conditions. Under the former conditions, the antibacterial effect is attributed to the acidic nature of the carbon, which lowers external pH, causing a drop in the intracellular pH of bacteria and thus leading to membrane disruption and death. The enhancement of photocatalytic disinfection efficiency under VL could be attributed to the generated holes in the carbon matrix. Surface chemistry plays a critical role and governs disinfection properties. The presence of strongly acidic groups, especially the sulfonic group, contributes to its bactericidal activity under dark conditions. Moreover, sulfur species, such as sulfones, sulfoxides, and sulfonic groups, contribute to hole generation under visible light. This study offers new insights into sulfur-doped carbons as metal-free materials for disinfection technologies.

## Supplementary Material



## Notes

Spectra of the light source, calibration curve of sulfur concentration using ICP-MS, calibration curve of sulfate concentration using IC, TEM images of C-1 and C-2, fluorescence images of *E. coli*, images of agar plate results, effect of C-2 on *Staphylococcus epidermidis*, temperature of the sample suspension during 1 h illumination, Mott–Schottky plot of C-2, EPR spectra obtained from C-2 containing spin label TEMPO after exposure to visible light (PDF)
