# Supplementary material for: Importance of S‑Doped Porous Carbon Acidity and Visible Light Photoactivity for Its Antibacterial Activity
Source: ACS Appl Mater Interfaces. 2025 Nov 4;17(47):64106–17. doi: 10.1021/acsami.5c14239 (PMC12673528; doi:10.1021/acsami.5c14239)
Supplement: Supplementary file 1 [file am5c14239_si_001.pdf]

Supporting information

Importance of S-doped porous carbon acidity and visible light photoactivity for its antibacterial activity

Danela Sadikaj[a], Abanob Fekri[b], Isabelle Bautista[b], Nafisatu Davis[c], Tasnim Agha Alkla[c], Muhammad Moueed Haider Mirza[b], Jiaying Wang[d], Phillip Stallworth[e], Natalie Hudson-Smith[b], Teresa J. Bandosz[f], Xiaojun Yu[d], Steven Greenbaum[e], Wanlu Li[c,g]*

a. Department of Earth and Environmental Science, Montclair State University, Montclair, NJ 07043, United States
b. Department of Chemistry, Saint Peter's University, Jersey City, NJ 07306, United States United States
c. Department of Chemistry and Biochemistry, Montclair State University, Montclair, NJ 07043, United States
d. Department of Biomedical Engineering, Stevens Institute of Technology, Hoboken, NJ 07030, United States
e. Department of Physics and Astronomy, Hunter College, New York, NY 10065, United States
f. Department of Chemistry and Biochemistry, City College of New York, New York, NY 10031, United States
g. New Jersey Center for Water Science and Technology, Montclair State University, Montclair, NJ 07043, United States

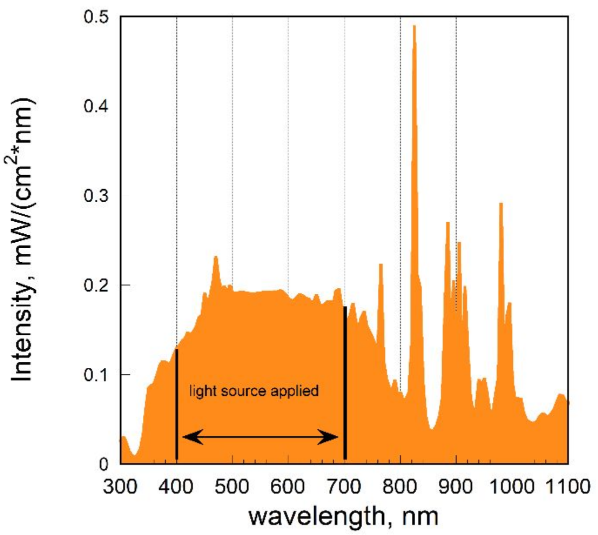

Figure S1. Spectra of the light source applied to the carbon samples.

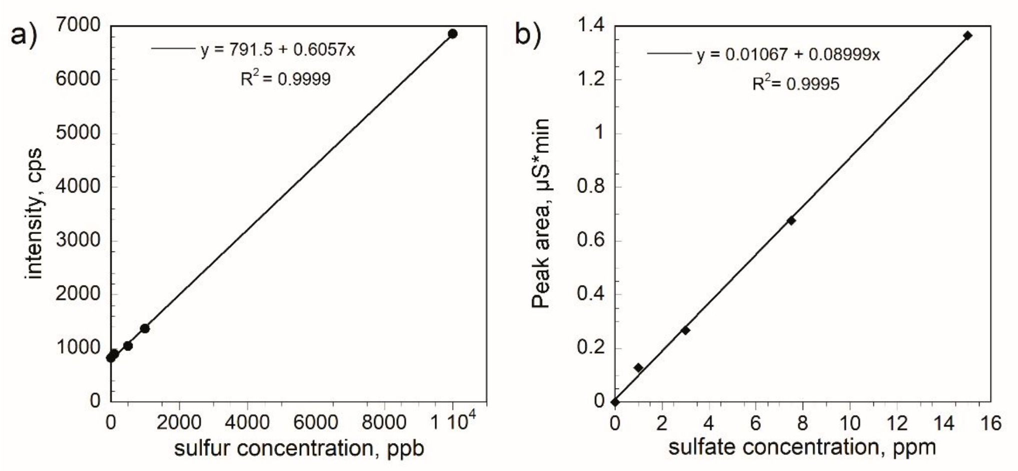

Figure S2. a) Calibration curve of sulfur concentration using inductively coupled plasma mass spectrometer, b) calibration curve of sulfate concentration using ion chromatograph.

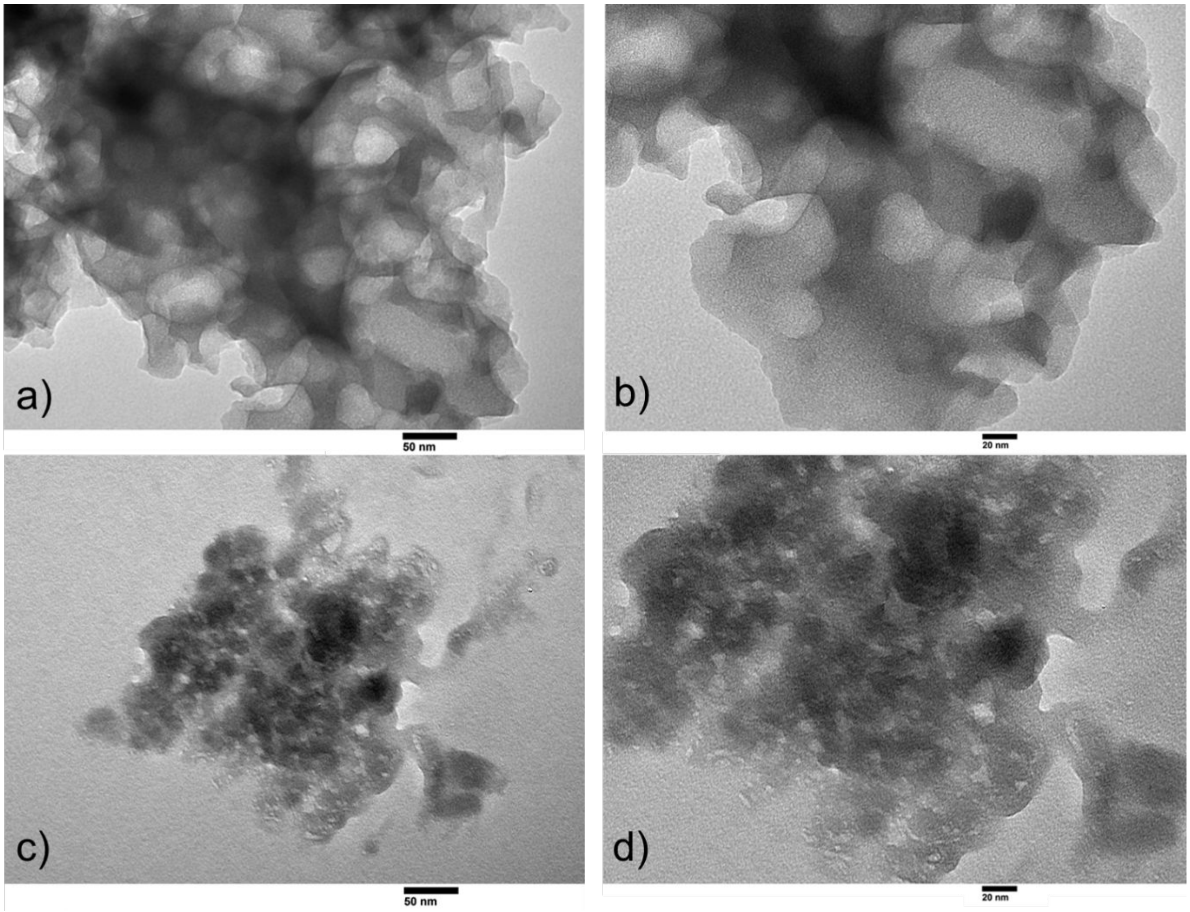

Figure S3. a, b) TEM images of C-1, and c, d) C-2 at different magnifications.

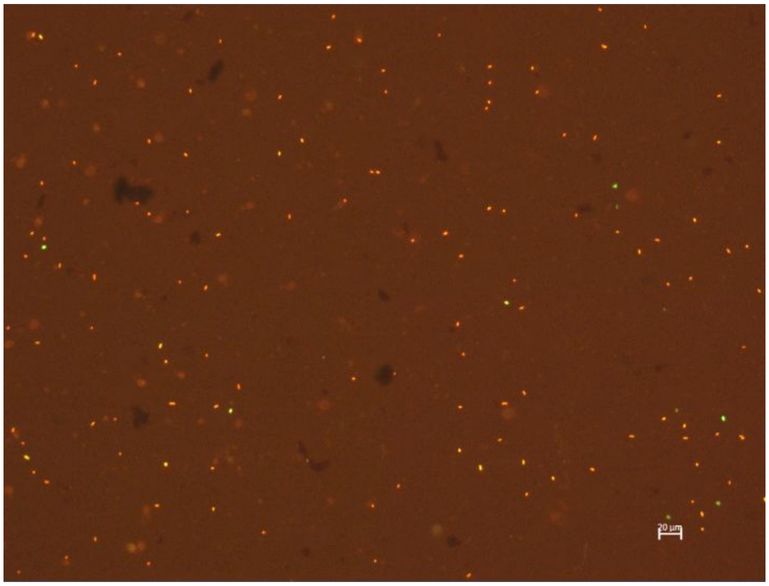

Figure S4. Fluorescence image of *E.coli* attachment on C-2 carbon under light.

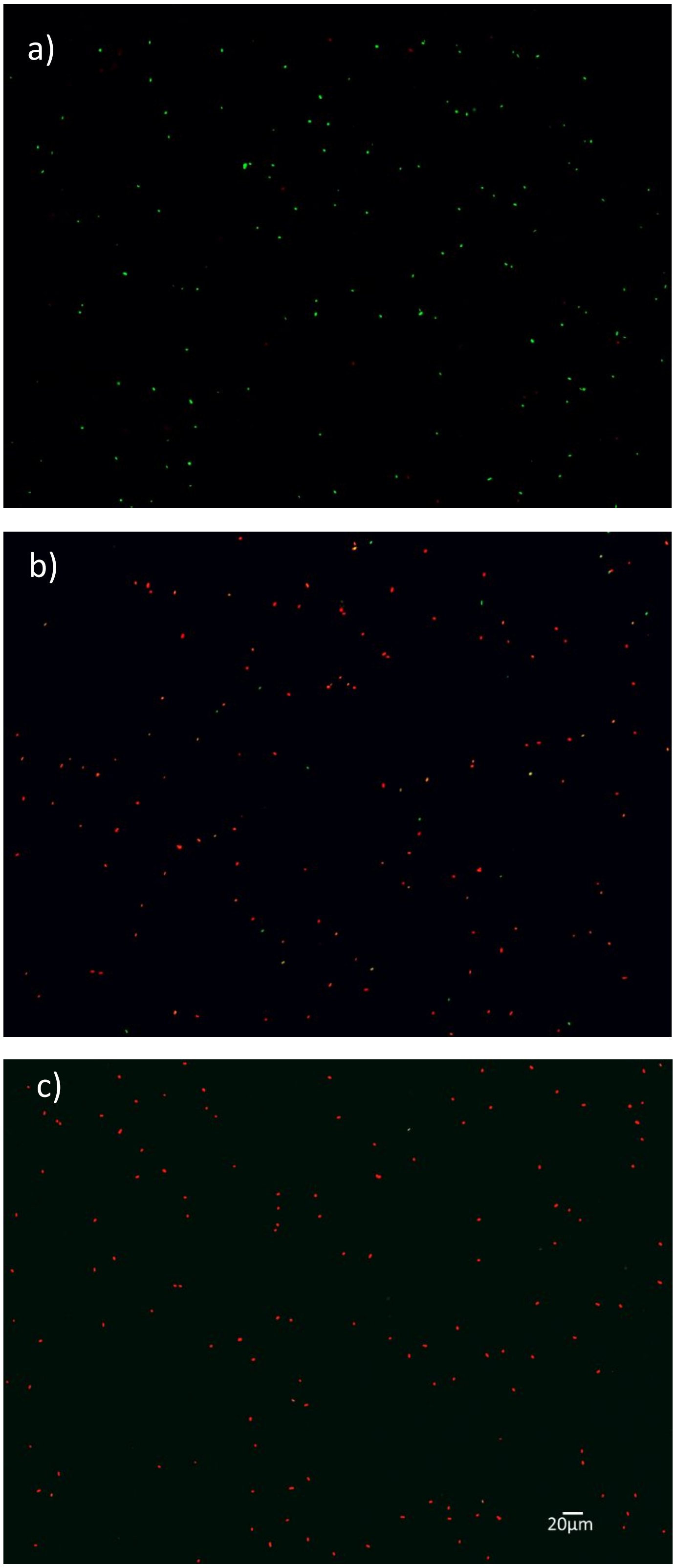

Figure S5. Original fluorescence images of live (green) and dead (red) cells of a) control, b) C-2 dark, and c) C-2 light. (Scale bars: 20 μm.)

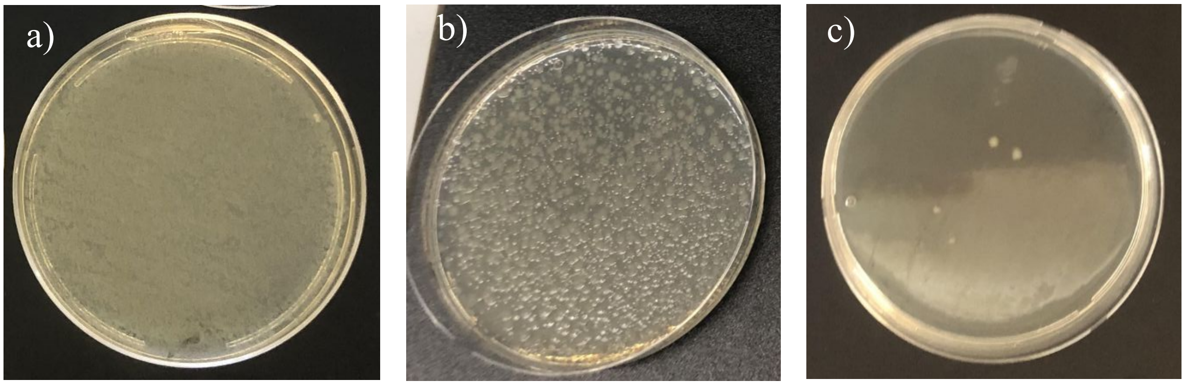

Figure S6. Images of agar plate results of antibacterial test with all suspensions (200uL) of a) control light-1h, b) C2-dark-1h, and c) C2-light-1h. ‘

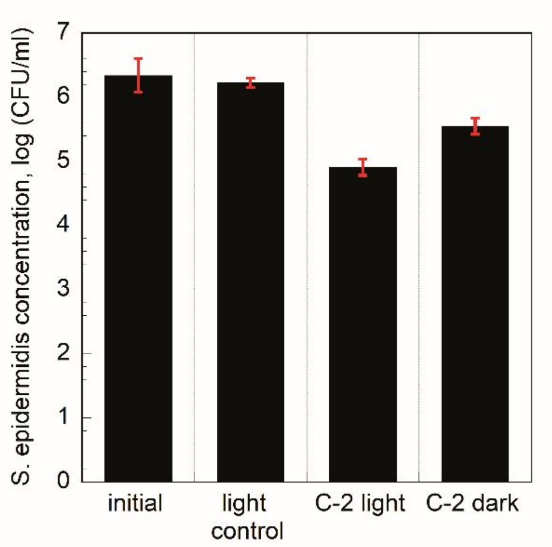

Figure S7. Effect of C-2 on Staphylococcus epidermidis (*S. epidermidis*) concentration under various conditions assessed by the colony counting method. (Cultured *S. epidermidis* cells treated under visible light in the isotonic saline are named as light control.)

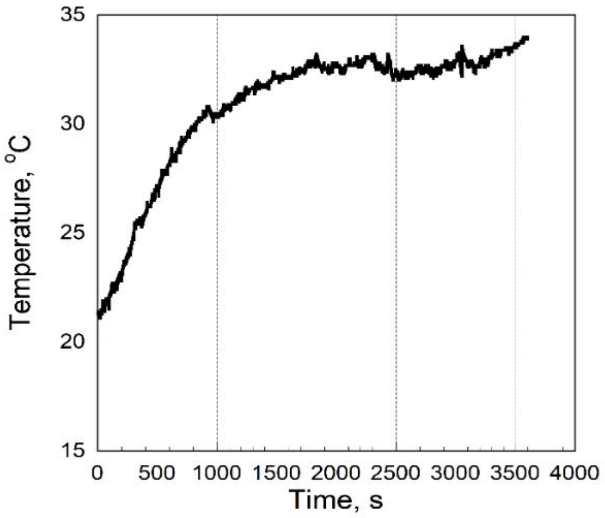

Figure S8. Temperature of the sample suspension during one-hour illumination.

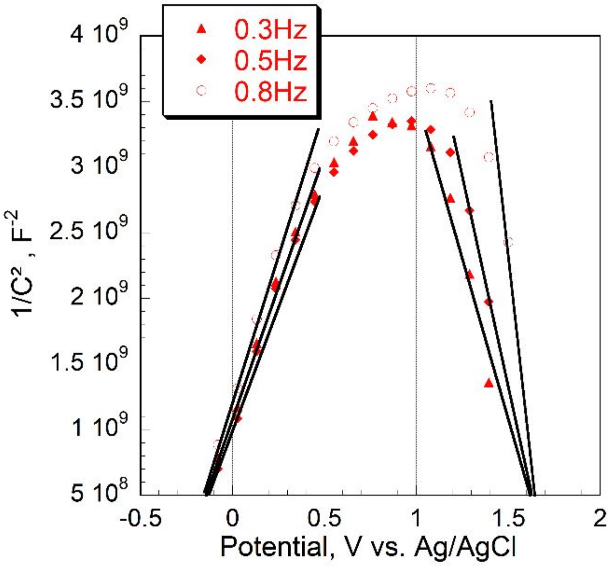

Figure S9. Mott-Schottky plot of C-2.

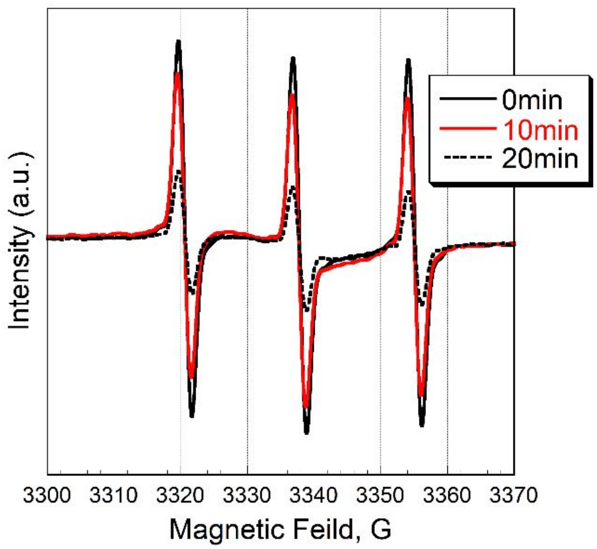

Figure S10. EPR spectra obtained from C-2 containing spin label TEMPO after exposure to visible light for 10min and 20min.
